# Phytochemicals and Cytotoxicity of *Quercus infectoria* Ethyl Acetate Extracts on Human Cancer Cells

**DOI:** 10.21315/tlsr2020.31.1.5

**Published:** 2020-04-07

**Authors:** Wan Nur Suzilla Wan Yusof, Hasmah Abdullah

**Affiliations:** School of Health Sciences, USM Health Campus, Universiti Sains Malaysia, 16150 Kubang Kerian, Kelantan, Malaysia

**Keywords:** *Quercus infectoria* extracts, Phytochemical constituents, Cytotoxic activity, Cytoselective, DNA fragmentation, Ekstrak *Quercus infectoria*, Kandungan fitokimia, Aktiviti sitotoksik, Ciri sitoselektif, Fragmentasi DNA

## Abstract

Conventional and modern cancer treatment were reported to manifest adverse effects to the patients. More researches were conducted to search for selective cytotoxic agent of plant natural product on cancer cells. The presences of wide range phytochemicals in *Quercus infectoria* (QI) extract have been implicated with the cytotoxic effect against various types of cancer cell which remain undiscovered. This present study aimed to evaluate cytotoxic effect of QI extracts on selected human cancer cells and then, the most potent extract was further analysed for general phytochemical constituents. QI galls were extracted successively with n-hexane, ethyl acetate and methanol yielded three main extracts; n-hexane (QIH), ethyl acetate (QIEA) and methanol (QIM), respectively. The most potent extract was qualitatively analysed for the present of tannin, alkaloids, glycosides, saponins, terpenoids, flavonoids and phenolic compounds. Next, the extracts were tested to determine the cytotoxic activity against cervical cancer cells (HeLa), breast cancer cells (MDA-MB-231) and liver cancer cells (Hep G2) using MTT assay. Cytotoxic activity of QI extracts against normal fibroblast (L929) cell line was also evaluated to determine the cytoselective property. Meanwhile, DMSO-treated cells served as negative control while cisplatin-treated cells served as positive control. The most potent extract then chosen to be further investigated for DNA fragmentation as hallmark of apoptosis using Hoechst staining. Qualitative phytochemical analysis revealed the presence of tannin, alkaloids, glycosides, saponins, terpenoids, flavonoids and phenolic compounds. QIEA extract exhibited the most potent cytotoxic activity against HeLa cells with (IC_50_ value = 6.33 ± 0.33 μg/mL) and showed cytoselective property against L929 cells. DNA fragmentation revealed QIEA induced apoptosis in the treated cells. The richness of phytochemical constituents in QIEA extract might contribute to the potency of cytotoxic activity towards HeLa cells.

## Abstract (ms)

Rawatan kanser secara moden dan tradisional dilaporkan telah memberi kesan sampingan kepada pesakit. Banyak kajian telah dijalankan untuk mencari agen sitotoksik yang hanya mensasarkan sel kanser menggunakan produk semulajadi daripada sumber tumbuh-tumbuhan. Kewujudan bahan fitokimia yang pelbagai dalam ekstrak *Quercus infectoria* (QI) memberi kesan toksik terhadap beberapa jenis sel kanser yang belum terungkai. Kajian ini bertujuan untuk menilai aktiviti sitotoksik beberapa jenis ekstrak dan memilih ekstrak yang paling poten untuk dianalisa bahan fitokimia secara umum. Ketuat QI diekstrak secara berperingkat menggunakan n-heksana, etil asetat dan methanol menghasilkan ekstrak: n-heksana (QIH), etil asetat (QIEA) dan methanol (QIM) masing-masing. Ekstrak yang paling poten dianalisis secara kualitatif untuk menentukan kehadiran tanin, alkaloids, glikosida, saponin, terpenoid, flavonoid dan sebatian fenolik. Seterusnya setiap ekstrak diuji aktiviti sitotoksik terhadap sel kanser servik, (HeLa), sel kanser mamari (MDA-MB-231) dan sel kanser hati (Hep G2) menggunakan cerakinan MTT. Aktiviti sitotoksik ekstrak QI juga diuji ke atas sel fibroblast normal (L929) untuk menentukan ciri selektifnya. Sel yang dirawat dengan DMSO bertindak sebagai kawalan negatif, manakala sel yang dirawat dengan sisplatin bertindak sebagai kawalan positif. Ekstrak yang paling poten seterusnya dipilih untuk menyelidik fragmentasi DNA sebagai tanda berlaku apoptosis menggunakan pewarnaan Hoechst. Analisis kualitatif menunjukkan kehadiran tanin, alkaloids, glikosida, saponon, terpenoids, flavonoid dan sebatian fenolik. Ekstrak QIEA adalah paling poten terhadap sel HeLa (IC_50_ = 6.33 ± 0.33 μg/mL) dan menunjukkan ciri sitoselektif terhadap sel L929. Fragmentasi DNA membuktikan QIEA mengaruh apoptosis dalam sel yang dirawat. QIEA yang kaya dengan bahan fitokimia kemungkinan menyumbang kepada kepotenan aktiviti sitotoksik terhadap sel HeLa.

**Highlights**

- Qualitative phytochemical analysis revealed the presence of tannin, alkaloids, glycosides, saponins, terpenoids, flavonoids and phenolic compounds.
- QIEA extract exhibited the most potent cytotoxic activity against HeLa cells with (IC_50_ value = 6.33 ± 0.33 μg/mL) and showed cytoselective property against L929 cells
- The cytotoxicity of QIEA extract has exerted DNA fragmentation in treated HeLa cells as hallmark of apoptosis.

## INTRODUCTION

Cancer is considered as the second cause of death around the world. Cancer is characterised by the abnormality of cell growth resulted in the uncontrolled multiplication of the normal cells to form tumors which in further invades into nearby parts of the body (Gandhiappan & Rengasamy 2012). Among all cancer types, cervical, breast and liver are among the 10 most recorded cancer cases worldwide (Bray *et al.* 2018).

Chemotherapy is the leading approaches in cancer therapies (American Cancer Society 2015) that involves the usage of chosen medications to control the metastasis of cancer cells (Caley & Jones 2012; Massague & Obernauf 2016). However, the drugs used for chemotherapy were reported to manifest adverse effect towards non-cancerous calls (Nurgali *et al.* 2018). Hence, there is an urge for the development of new anticancer medications targeted exclusively on cancer cells. In this regards, natural products from plants are expected to produce candidates for the development of new targeted anticancer drugs. It has been estimated that about 60% of modern drugs were derived from natural origin (Hanahan & Weinberg 2000; Gandhiappan & Rengasamy 2012).

*Quercus infectoria* Olivier (Fagaceae) or also known as galls of *Quercus infectoria* (QI) is a small tree found in Greece, Asia Minor and Iran (Kottakkal 1995). The main constituents found in the galls of QI are tannin (50%–70%) and small amounts of free gallic acid and ellagic acid (Kottakkal 1995). QI galls locally known as “manjakani” was claimed to be highly beneficial for the Malay Kelantanese postpartum women (Bhattacharjee 2001). There were no reports regarding hazardous effects from the past uses of this herbal preparation. QI galls aqueous extract showed high potential in skin whitening and antioxidant properties as the extract inhibited the superoxide and DPPH radical scavenging activities, and tyrosinase activities (Borgia *et al*. 1981).

Studies indicated that QI galls have a variety of pharmacological properties including being an astringent (Dar *et al*. 1976), antidiabetic (Kaur *et al.* 2007), antitremorine local anesthetic, antiviral (Harborne 1986), potential antibacterial (Hwang *et al*. 2000), antifungal (Baharuddin *et al.* 2015; Magbool *et al.* 2018), larvicidal (Ikram & Nowshad 1977) and anti-inflammation (Mc Clure 1975; Muhamad & Mustafa 1994). Various types of QI extracts were reported to poses anticancer activity on cervical cancer cells (Hasmah *et al*. 2010) and colon cancer cells (Roshni & Ramesh 2013). The main constituents found in QI extracts are tannin and become a major source of gallic and tannic acid (Ong & Nordiana 1999). The presences of various compounds such as flavonoids, polyphenolics, tannins and steroid have been implicated in a number of medicinal properties of the plants (Yoo *et al.* 2018). Thus, this present study was intended to determine the cytotoxic effects of QI extracts and then to analyse the general phytochemical constituents of the most potent extract towards the most sensitive cancer cell lines.

## MATERIAL AND METHODS

### Plant Materials

Galls of *Quercus infectoria* (QI) was obtained from Chinese herbal outlet in Kota Bharu, Kelantan, Malaysia. To ensure the correct species of the galls used, organoleptic properties investigation based on morphological appearance included external colour, odour, size, surface and texture was conducted as described by Asif *et al.* (2012). Prior to examination of morphological appearance, the same batch of the galls were previously analysed macroscopically and microscopically involving outer and inner part of the plant materials (Hasmah *et al.* 2010). The confirmed of plant materials were proceeded for plant extraction.

### Plant Extraction

The QI galls were processed to become powder form and then was successively extracted with organic solvent (n-hexane, ethyl acetate and methanol) by soaking method. Amount of 50 g QI galls powder was homogenised in 1 L beaker containing 200 mL n-hexane and placed in water bath for 24 h with constant temperature at 50°C. After that, the extracts solution was filtered and concentrated by rotary evaporator. The crude extracts then were lyophilised in freeze-drier and stored at −20°C prior used (Fatima *et al*. 2001). The procedures were repeated for the other solvents using the same materials successively and yielded n-hexane extract (HE), ethyl acetate extract (EA) and methanol extract (ME). All extracts were tested against cell cytotoxicity assay.

### Phytochemical Screenings of QI Extract

The major groups of phytochemical contents in the extracts were screened qualitatively to determine the presence of alkaloids, tannins, glycoside, flavonoids, terpenoids, saponins and phenolic compounds. The experiments were conducted based on relevant previous study with some modifications.

#### Alkaloids

Few drops of Mayer’s reagent were added to the extract; cream colour precipitate indicates the presence of alkaloids (Rohana *et al.* 2004).

#### Tannins

1 mL of 5% FeCI_3_ was added to the extract, presence of tannin was indicated by the formation of bluish black or greenish black precipitate (Rohana *et al.* 2004).

#### Glycosides

2 mL of glacial acetic acid, few drops of 5% FeCI_3_ and concentrated H_2_SO_4_ were added to the extract. Reddish brown color at the junction of two liquid layers and upper layer appears bluish green indicates the presence of glycosides (Siddiqui & Ali 1997).

#### Flavonoids

Few drops of 10% concentrated sulphuric acid was added to the extract, followed by 1 mL ammonia, formation of greenish yellow precipitate indicates the presence of flavonoids (Rohana *et al.* 2004).

#### Terpenoids

5 mL chloroform and 2 mL concentrated sulphuric acid was added into 2 mL extract. Reddish brown colorations of interface indicate the presence of terpenes (Soon & Hasni 2005).

#### Saponins

20 mL water was added to 150 mg extract and shaken vigorously; layer of foam formation indicates the presence of saponins (Rohana *et al.* 2004).

### Cell Culture

Different types of cancerous and non-cancerous cells were used in this study, namely, cervical cancer (HeLa), breast cancer (MCF-7 and MDA-MB-231), liver cancer (Hep G2), and normal fibroblast (L929). These cell lines were purchased from American Type Culture Collection (ATCC, Manassas, Virginia, USA). The development medium utilised was Dulbecco’s Modified Eagle Medium (DMEM) (Invitrogen, USA) supplemented with 10% Fetal Bovine Serum (FBS) (Gibco, Life Technologies, USA) and 1% penicillin-streptomycin (Invitrogen, USA).

Cryovial containing frozen cells was thawed by gentle agitation in 37°C water bath. Then, the cryovial contents were transfer to a centrifuge tube containing 10 mL growth medium and spinned at 125 xg for 5 min. The supernatant was discarded and cells pellet was gently re-suspended in complete growth medium and dispensed into a 25 cm^3^ sterile culture flask (Nunc, Denmark). The cell culture was maintained in 37°C humid incubator with 5% (v/v) CO_2_. All procedures were performed under controlled aseptic conditions.

### MTT Cytotoxicity Assay

The cytotoxicity assay was performed using MTT assay as previously described with some modifications. Briefly, cells were seeded for 24 h prior to treatment in 96-well plate at 5 × 10^4^ cells/well in order to obtain 80% confluent cultures. The extract was dissolved in DMSO (Sigma Chemical Co., St. Louis, Missouri, USA) and added to the culture medium. Cells were treated with QI extract and cisplatin in concentration ranging from 0–99 μg/mL. Control cultures received the same concentration of DMSO. Plated and treated cells were incubated for 72 h at 37°C in a humidified atmosphere with 5% CO_2_. At the end of incubation periods, 50 μL of MTT solution (2 mg/mL MTT in plain culture medium; Sigma Chemical Co.) was added to each well. The plate was then incubated for 4 h. After the periods, MTT solution was removed and the purple formazan crystal formed at the bottom of the wells was dissolved with 200 μL DMSO for 20 min. The absorbance was read at 570 nm on a micro plate reader.

### Mechanism of Cell Death

Cell cytotoxicity was featured by cell death mechanism. The most potent extract found from the cytotoxicity assay was further studied for mechanisms of cell death. To confirm the cell death by apoptosis, the nuclear morphological changes of the treated cells was observed using Hoechst 33258 stain. Cells with 80% confluence were washed with PBS and cells trypsinised with 0.25% (v/v) trypsin-EDTA. Next, 5 × 10^4^ cells/ml of cells were cultured in new 25 cm^3^ culture flask and incubated in 37°C with 5% CO_2_ incubator. The confluence cells were treated with the most potent extract and cisplatin for 24, 48 and 72 h respectively. Untreated cells were utilised as negative control and cells treated with cisplatin served as positive control.

After the treatment hours, cells were trypsinised and centrifuged at 300 xg for 5 min. The supernatant was discarded and cells pellet was dissolved in 10 μL PBS. Next, cells suspensions were smeared on poly-L-lysin slides and air-dried. Then, 10% (w/v) paraformaldehyde was added to fix the cells to the slides. The cells were permeabilised with 0.2% (v/v) Triton-X for 1 min at room temperature. Lastly, cells were stained with 30 μg/mL DNA color Hoechst 33258 stain for 30 min at room temperature and viewed under fluorescence microscope (Zeiss) at magnification 40x.

### Statistical Analysis

Data were expresses as mean ± SEM of three independent experiments. Data analysis were performed using Statistical Package of Social Science (SPSS) Software version 20. The Shapiro-Wilk test was used for normality. The statistical significances of differences were determined using one-way analysis of variance (ANOVA) followed by Bonfferoni test and probability values of *p* < 0.05 was considered to be statistically significant.

## RESULTS AND DISCUSSION

### Organoleptic Properties of *Quercus infectoria* (QI) Galls

**Figure 1:**
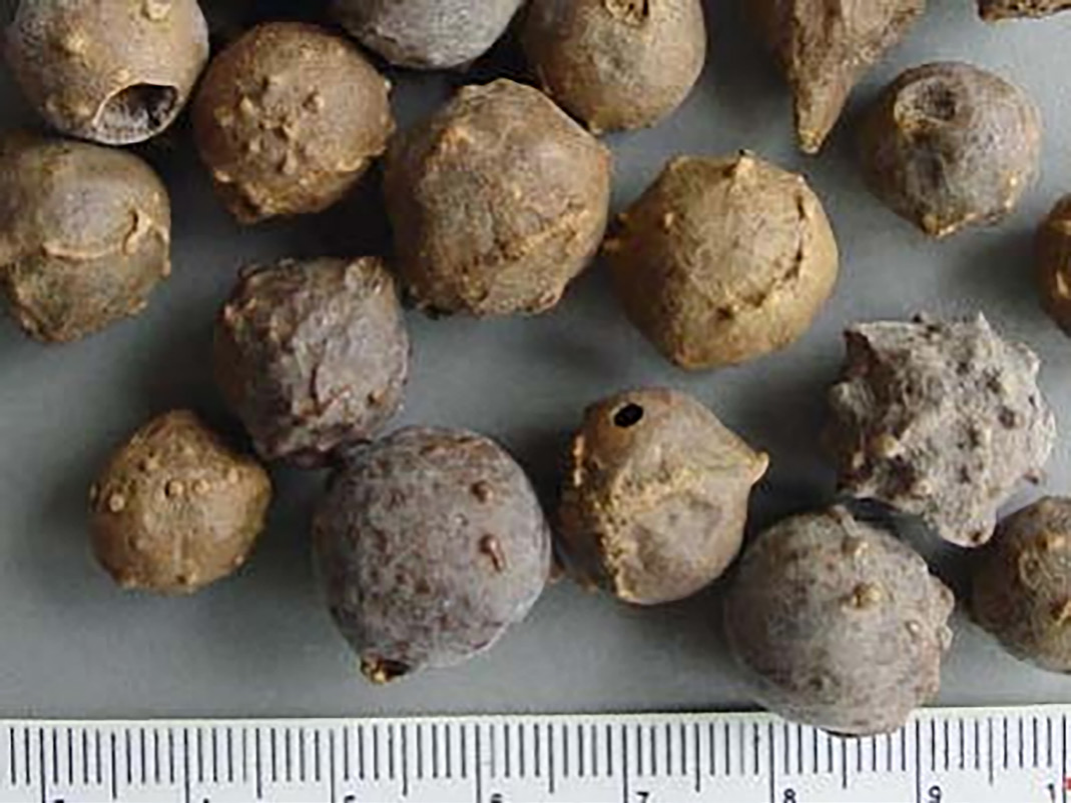
Organoleptic properties of QI galls.

The parameters of organoleptic properties of QI galls investigated were external colour, size, surface, texture and odour. All of these parameters are important as the morphological identification (Fig. 1). Morphology of the QI galls used in this study exhibited similar properties (Table 1) as described by Asif *et al*. (2012).

### Phytochemical Screening

The medicinal values of plant lies on bioactive phytochemical constituents of the plant which shows various physiological effects for human body. Hence, phytochemical screening is a tools to elucidate important compound which could be based of modern drugs for curing various diseases (Azad *et al*. 2013) even though latest trends utilise high-throughput screens based on molecular targets which had led to a demand for the generation of large libraries of compounds (Newman & Cragg 2016).

Qualitative phytochemical screening is an essential step towards discovery of new drugs as it provides the information regarding the presence of a particular primary or secondary metabolites in the plant extract of clinical significance (Trease & Evan 1989). Based on the qualitative analysis, the phytochemical evaluation of QIEA extract revealed the presence of tannins, alkaloids, saponins, terpenes, flavonoids, glycosides and phenolic compounds (Table 2).

Our finding was in accordance with previous study which revealed the presence of diverse groups of compounds including saponins, alkaloids, tannins, glycosides, triterpenes, sterols, phenolic mixes, starches and flavonoids in various extracts of QI galls (Shrestha *et al.* 2014). Furthermore, previous study also documented variation of gallic acid and tannic acid distribution in various QI galls extracts. Abdullah *et al.* (2017) reported that polyphenolic compounds constituted by gallic acid derivatives and hydrolysable tannins served as major phytoconstituents present in the QI extract analysed by MS/MS.

The cytotoxic activity possessed by QIEA extract may also be mediated by the unique combination of phytochemicals in the extract (Saxena *et al.* 2013). The cytotoxic activity of phytochemicals in QIEA extract have been documented in some articles (Kanadaswami *et al*. 2005; Okuda & Ito 2011; Lu *et al*. 2012). For example, gallotannic acid (tannin) which present as real constituent of QI galls has been uncovered to show antimutagenic, anticancer and cancer prevention agent properties (Srivastava *et al.* 2000; Gao *et al.* 2018).

### Cytotoxicity Activity of QI Galls Extract

The effects of QI galls n-hexane, ethyl acetate and methanol extracts on cells proliferation were determined from IC_50_ value. The IC_50_ value is the concentration of the extract or anticancer agent required to inhibit 50% of cells population (Lim *et al*. 2009). Extract that showed the best inhibition on the tested cancer cell lines, represented by the lowest IC_50_ value (less than 20 μg/mL) following 72 h treatment was selected as the most potential extract (Zakaria *et al*. 2009).

According to the results, the IC_50_ values calculated in response to QIH treatment for HeLa, MCF-7, MDA-MB-231 and Hep G2 cell lines were in descending cytotoxic activity 47.5 ± 0.58 μg/mL, 49.8 ± 1.46 μg/mL, 95.7 ± 2.51 μg/mL and 97.4 ± 0.88 μg/mL, respectively. In addition, the IC_50_ values in descending cytotoxic activity after treated with QIEA extract was 6.33 ± 0.33 μg/mL for HeLa, while for MCF-7 and Hep G2 were 20.5 ± 1.23 μg/mL and 23.6 ± 2.14 μg/mL, respectively. For MDA-MB-231, QIEA extract showed very low cytotoxicity as the IC_50_ value was ≥ 99 μg/ml. Besides that, the IC_50_ values obtained after treated with QIM extract was 23.8 ± 0.91 μg/mL for HeLa, while for MDA-MB-231 and Hep G2 were 90.2 ± 0.89 μg/mL and 85.1 ± 0.34 μg/mL respectively. It was found that, QIM extract showed very low toxicity againts MCF-7 cell line as the IC_50_ value obtained was ≥ 99 μg/mL. Within all tested cancer cells, it was demonstrated that QIEA extract exhibited best cytotoxic activity againts Hela cell line as the IC_50_ value obtained was the lowest and ≤ 20 μg/mL.

After incubation of cell lines with QIEA extract for 72 h, the extracts obviously showed cytotoxic effects towards HeLa and MDA-MB in concentration dependent manner. The EA extract exerted higher cytotoxicity effect towards HeLa cells with IC_50_ of 6.33 ± 0.33 μg/mL. However, the QIEA extract was less active against MDA-MB cell line as the IC_50_ value was 90.0 ± 16.9 μg/mL. The extract showed no cytotoxic effect towards normal cells at IC_50_ concentration that inhibit the growth of HeLa cells. Moreover, QIEA exhibited cytotoxic activity towards MCF-7 and Hep G2. Previously QIEA extract exerted cytotoxic activity towards ovarian cancer cells, Caov-3 (Hasmah *et al*. 2010). QIEA exhibited no cytotoxic effect againts normal fibroblast (L929) cell line. The ability to kill cancer cells without affecting normal cells reflects the cytoselective property of QI galls extracts. A survey on traditional usage of QI reported no side effect after the consumption of it herbal preparations (Soon *et al*. 2007). The other study on non-cancerous ovarian (CHO) and normal kidney (Vero) cells also demonstrated no cytotoxic effect of QI galls (Ismail *et al*. 2010). Recently, Hazwani *et al*. (2018) reported moderate cytotoxicity activity exerted by *Q. infectoria* aqueos extract and *Q. infectoria* vaginal cream against HeLa cell with IC_50_ values of 13.90 ± 2.27, and 20.80 ± 1.94, respectively. Both preparation exerted high DPPH radical scavaging activity.

In this study, the cytotoxic effects of QIH, QIEA and QIM extracts againts normal cell line were also investigated. All extracts showed low cytotoxicity activity (IC_50_ ≥ 20 μg/mL) on normal fibroblast (L929) cell line (Table 3).

The most widely used and commercial anticancer drug, cisplatin was used as positive control (Florea & Büsselberg 2011, Hazwani *et al.* 2018). Cisplatin-treated HeLa cell showed lowest IC_50_ value (10 ± 0.67 μg/mL), followed by MDA-MB-231 (11.8 ± 0.67 μg/mL), Hep G2 (14.6 ± 0.34 μg/mL) and MCF-7 (16.9 ± 3.53 μg/mL). However, no significance difference (*P* > 0.05) was observed within the tested cancer cell lines. The cytotoxic activity screening towards normal fibroblast (L929) cell lines demonstrated high cytotoxic activity of cisplatin with IC_50_ value obtained was 18.7 ± 5.73 μg/mL. This showed that cisplatin was wellknown not cytoselective agent as it inhibits the proliferation of both cancerous and non-cancerous cells. Cisplatin was used as positive control and the IC_50_ values against HeLa cell lines was 10 ± 0.67 μg/mL. The cytotoxic activities of the extracts and cisplatin were varying in three cancer cell lines tested. In the US NCI plant screening program, a crude extract is generally considered to have *in vitro* cytotoxic activity if the IC_50_ value (concentration that cause a 50% cell killed) in carcinoma cells, following incubation between 48 and 72 h, is less than 20 μg/mL, while it is less than 4 μg/mL for pure compounds (Umachigi *et al.* 2008).

Cisplatin is a broad range of anticancer drugs used for chemotheraphy. The current study revealed the inhibitory properties towards tested of cancer cell lines with potent IC_50_ less than 20 ug/mL (Umachigi *et al*. 2008). However, cisplatin also inhibited the growth of normal cells as well. The non-selective cytotoxic activity of cisplatin is in line with nephrotoxicity effects post chemotherapy as reported previously (Desoize & Madoulet 2002; Florea & Büsselberg 2006; Shah & Dizon 2009; Günes *et al*. 2009; Tsang *et al*. 2009).

Interestingly, QIEA showed the best IC_50_ value as it also demonstrated no cytotoxicity towards normal cells (Table 3). Hence, QIEA extract was selected for further investigations in this study.

### Mechanism of Cell Death

Cells undergoing apoptosis usually demonstrated morphological and biochemical components, including chromatin aggregation, nuclear and cytoplasmic condensation, and partition of cytoplasmic and nucleus into membrane-bound vesicles (Kerr *et al.* 1972). Chromatin condensation and nuclear fragmentation served as hallmarks for nuclear morphology of the apoptotic cells, which can be observed under fluorescence microscope through several techniques such as, DNA-binding stains, like 4′,6-diaminido-2-phenylindole, Hoechst and others (Ziegler & Groscurth 2004).

Those findings also demonstrated biochemical alterations that served as key players of the apoptotic mechanisms that are responsible of evasion from apoptosis and therefore of tumor development and resistance to therapies. Thus intensive investigation on molecular mechanisms of apoptosis in cancer cells has led to the identification of the several molecules involved in both the intrinsic and the extrinsic apoptotic pathways (Pistritto *et al*. 2016). The current study employed nuclear fragmentation as early hallmark of apoptosis, as therapeutic target prior to elucidation of apoptotic protein in HeLa cells treated QIEA in future research.

**Figure 2:**
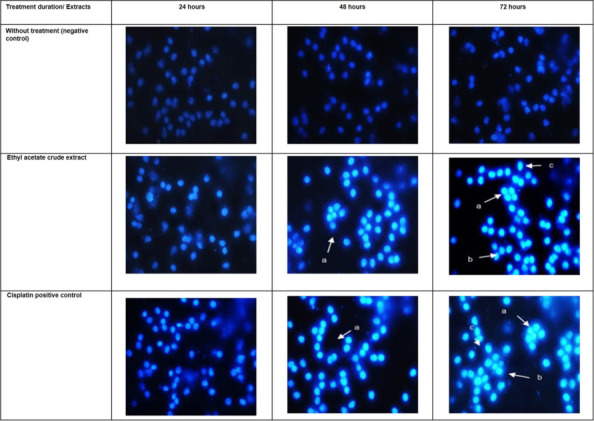
Nuclear morphological changes of HeLa cells stained with Hoechst 33258 stain; (a) nuclear fragmentation, (b) shrinkage of cell nucleus, (c) apoptotic bodies. Magnification: 40X, n = 3.

In the untreated HeLa cells, nuclei were rounded and homogenously stained with Hoechst 33258 stain in the period of 24, 48 and 72 h (Fig. 2). Since the cells were viable and did not demonstrated nuclear morphological changes, no fluorescence were emitted in the untreated HeLa cells.

After 24 h treated with QIEA extract, the morphology of apoptosis began to appear in HeLa cells. It was observed that fluorescence were emitted from the nuclear region of DNA which are the common features of apoptosis (Fig. 2). As the treatment period prolonged to 48 h, chromatin condensation and DNA fragmentation were much more visible (Fig. 2). After 72 h, small fluorescence masses were detected which indicated the presence of apoptotic bodies (Fig. 2).

In addition, the results also showed similar pattern for morphological changes in HeLa cells treated with cisplatin which served as positive control for same treatment period (Fig. 2).

Based on the observations, HeLa cells treated with QIEA, showed the common characteristics of apoptotic cell death such as chromatin and nuclear condensation, DNA fragmentations and formation of apoptotic bodies (Zakaria *et al.* 2009). Chromatin condensations and DNA fragmentations in HeLa cells were started to be visible after 24 h of QIEA treatment. After 48 h, it was observed that the fluorescence was brighter and became more apparent. This showed that, at later stage of apoptosis, the nuclei further condensed and fragmented with intact cell membrane (Majno & Joris 1995). Besides that, the formation of the apoptotic bodies which appeared as small fluorescence spots, further confirmed the occurence of apoptosis in response to 72 h of treatment. As compared to this treated group, it was showed that, no change in nuclear morphology was detected in untreated HeLa cells. This findings is in accordance with previous study by Hasmah *et al.* (2010), in which HeLa cells treated with QI galls ethanolic extract experienced similar apoptotic manifestations.

Meanwhile, the cisplatin-treated HeLa cells also showed nuclear fragmentation and nuclear condensation with similar pattern as observed in QIEA-treated HeLa cells, and this strengthen the current findings (Sedletska *et al.* 2005). Condensed nuclei with fragmented chromatin in the treated cells represented changes in mitochondrial matrix morphology which clearly indicated the role of mitochondria (Jaudan *et al.* 2018) as indicated in HeLa cells treated with Pinostrabin (P_N_) a naturally occurring dietary plant bioflavonoid. Induction of cytotoxic cell death through apoptosis in HeLa cells treated QIEA are possibly through DNA damage mechanism (Shang *et al.* 2016) exhibited by DNA fragmentation. However, further investigations are necessary to elucidate full DNA damage mechanism.

## CONCLUSION

As conclusion, QIEA extract possessed the most potent cytotoxic activity towards cervical cancer cells (HeLa) with the lowest IC_50_ value among all the tested extract. In addition, cytotoxic activity of QIEA towards normal fibroblast (L929) revealed the cytoselective effect. The cytotoxic activity of QIEA were regulated by apoptosis cell death evidenced by the DNA fragmentation and chromatin condensation in the treated cells. The manifestation of cytotoxic effect and cell death event were might be due to the present of unique range of phytochemicals in the extract including tannins, alkaloids, flavonoids, glucosides, saponins, terpenoids and phenolic compounds. Thus, the QIEA deserve further study to elucidate the phytochemicals entity and detail mechanism of cell death.

## Figures and Tables

**Table 1:** Organoleptic properties of QI galls.

No	Organoleptic characteristics	Result
1	External colour	Dark yellowish brownish
2	Odour	Astringent
3	Size	Small (diameter 1–1.5 cm)
4	Surface	Rough and horny
5	Texture	Hard and woody

**Table 2:** Phytochemical constituents in QIEA.

Phytochemical constituents	Observations	Results
Alkaloids	The formation of the cream color precipitate	Detected
Tannins	The formation of the bluish-black precipitate	Detected
Saponins	Layer of foam formed	Detected
Terpenoids	Reddish-brown color formed	Detected
Flavanoids	The formation of greenish-yellow precipitate	Detected
Glycosides	Reddish brown color between the liquid layer and upper layer turn color to bluish-green	Detected
Phenolic compounds	The formation of the blue-green precipitate	Detected

**Table 3:** The IC_50_ values for QI galls extracts and cisplatin against non-cancerous cell lines, *P* < 0.05 was taken as significantly different from positive control (cisplatin).

Extracts/compound	L929
QIH	> 99
QIEA	>99
QIM	98.3 ± 2.67
Cisplatin	18.7 ± 5.73
